# Commentary: LncRNA-T199678 Mitigates α-Synuclein-Induced Dopaminergic Neuron Injury via miR-101-3p

**DOI:** 10.3389/fnagi.2021.650840

**Published:** 2021-03-12

**Authors:** Youcui Wang, Xiaoqin Zhang, Fenghua Chen, Leilei Chen, Junxia Xie

**Affiliations:** Shandong Provincial Key Laboratory of Pathogenesis and Prevention of Neurological Disorders, Shandong Provincial Collaborative Innovation Center for Neurodegenerative Disorders, Institute of Brain Science and Disease, Qingdao University, Qingdao, China

**Keywords:** Parkinson's disease, lncRNA-T199678, miRNA-101-3p, dopaminergic neuron injury, α-Synuclein

## Introduction

A hallmark of pathological characteristics in Parkinson's disease (PD) is the presence of eosinophilic inclusion bodies, such as Lewy bodies (LBs), in the substantia nigra (SN). The major component of LBs is an aggregated α-synuclein (α-Syn) that leads to neurotoxicity (Bu et al., 2020a; Wang et al., 2020). Although it has been well-documented that misfolding and abnormal aggregation of α-synuclein (α-Syn) are closely associated with the progressive loss dopaminergic neurons (Bu et al., 2020a; Wang et al., 2020), underlying molecular mechanisms remain unclear. Increasing evidence suggests that the abnormal expression of long non-coding RNAs (lncRNA) is closely related to the pathogenesis of PD (Ni et al., 2017; Lin et al., 2018). In the *in vivo* and *in vitro* models of PD, it has been observed that lncRNAs regulate the expression and aggregation of α-Syn (Lu et al., 2018; Zhang et al., 2019; Sun et al., 2020). Notably, they were reported to have altered expression in the SN of the patients diagnosed with PD (Ni et al., 2017). Although the identification of this altered expression is essential in the patients for a better understanding of the underlying molecular mechanisms, there is little knowledge about the role of lncRNAs in the pathogenesis of PD.

## Overexpression of lncRNA-T199678 Alleviates α-Syn-Induced Injury in Cellular Model of PD by Targeting miR-101-3p

**Figure 1:**
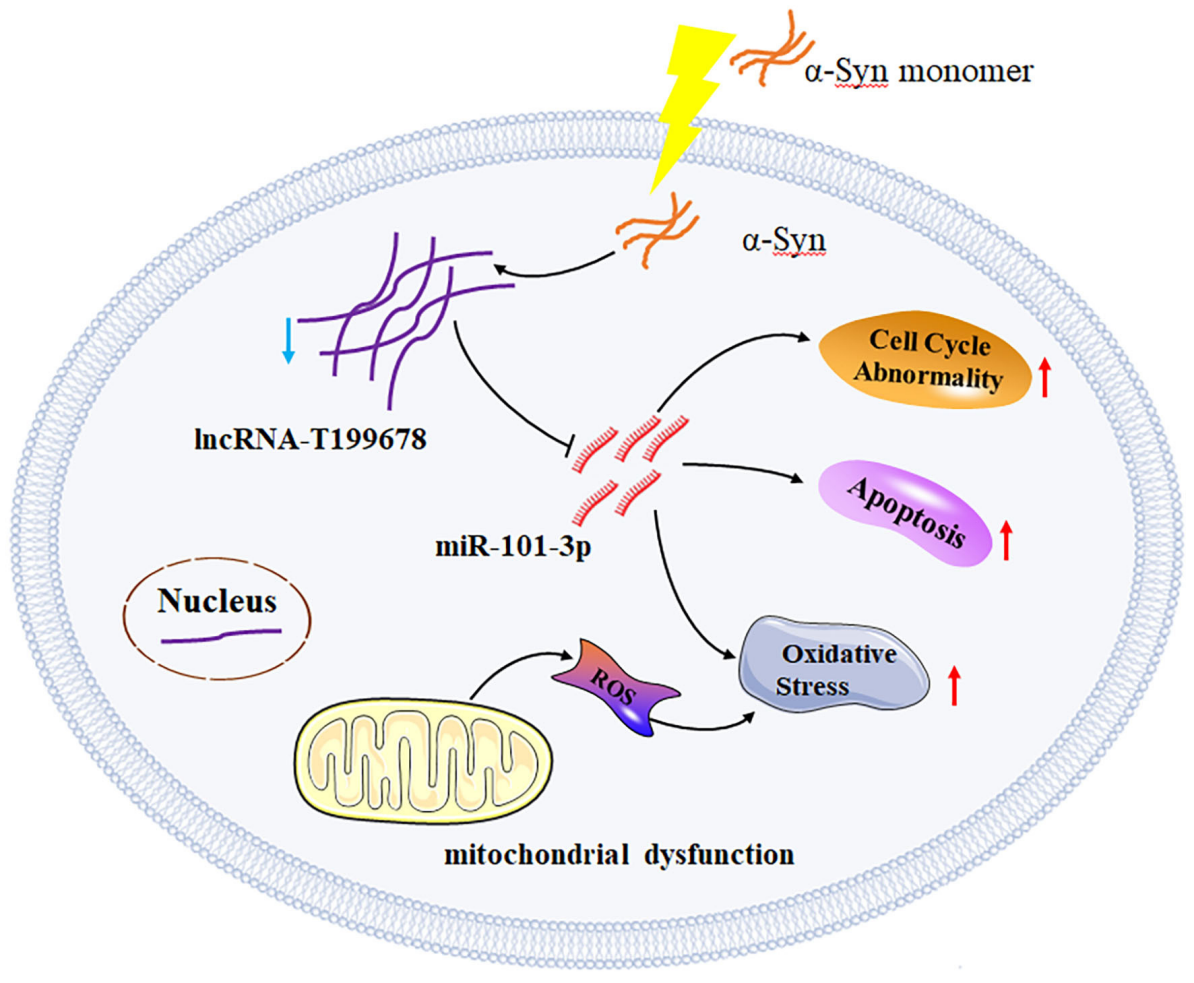
Overexpressed lncRNA-T199678 ameliorates α-Syn-induced neural damage via regulating intracellular oxidative stress, cell cycle dysfunction, and apoptosis by targeting miR-101-3p in the α-Syn-induced cellular model of PD (Bu et al., 2020b).

Previously, gene microarray analysis performed by Tao et al. revealed decreased expression of lncRNA-T199678 in an exogenous α-Syn-induced SH-SY5Y cellular model of PD suggesting that it may be involved in dopaminergic neuron loss (Lin et al., 2018). In this study (Bu et al., 2020b), the function of lncRNA-T199678 in α-Syn-induced dopaminergic neuron injury was further investigated. Given that the localization of lncRNA in the cell is associated with its function, Tao et al. first verified the presence of lncRNA-T199678 in the cytoplasm and a small amount in the nucleus of SH-SY5Y cells (Figure 1) (Bu et al., 2020b). In order to examine the effect of lncRNA-T199678 on the α-Syn-mediated dopaminergic neuron injury, LncRNA-T199678 overexpression or silencing cell lines was constructed in the α-Syn-induced SH-SY5Y cellular model of PD. Although the level of reactive oxygen species (ROS), the number of dividing cells, and the percentage of apoptotic cells, were significantly higher in the α-Syn-treated group and lncRNA-T199678 silencing groups, an overexpressed lncRNA-T199678 reversed intracellular oxidative stress, apoptosis, and abnormal cell cycle regulation indicating that it alleviated the damage from exogenous α-Syn (Bu et al., 2020b).

The striatum of patients with multiple system atrophy was observed to have an overexpressed miR-101 that was associated with α-Syn deposits, autophagy (Valera et al., 2017), inflammation (Han et al., 2019), oxidative stress, and apoptosis (Yi et al., 2019). In this study, the result indicated that miR-101-3p was a potential downstream molecular target of lncRNA-T199678 (Bu et al., 2020b). Further investigation revealed that an overexpressed lncRNA-T199678 inhibited α-Syn-induced neuronal damage via regulating intracellular oxidative stress, cell cycle dysfunction, and apoptosis that was reversed by the miR-101-3p mimic (Figure 1) (Bu et al., 2020b). Therefore, an overexpressed lncRNA-T199678 ameliorated α-Syn-induced dopaminergic neuron injury by targeting miR-101-3p. This study highlighted the role of lncRNA-T199678 in cellular models of PD and suggested new potential molecular targets for PD.

## Discussion

Mounting evidence has shown that lncRNAs are involved in regulating neuroinflammation, oxidative stress (Cai et al., 2020), apoptosis (Zhang et al., 2020), and autophagy (Yan et al., 2018) that are related to the pathogenesis of PD. Differentially expressed lncRNAs were identified in the circulating leukocytes of patients with PD as well as healthy subjects (Fan et al., 2019). Additionally, the expression of lncRNA MEG3, that is involved in the aggravation of non-motor symptoms, cognitive decline, and PD stage, was downregulated in the plasma of PD patients as compared to healthy controls (Quan et al., 2020), whereas lncRNA NEAT1 was observed to be upregulated in peripheral blood cells of PD patients (Boros et al., 2020). Therefore, investigations into the altered expression of lncRNAs is beneficial for better understanding as well as developing new therapeutic targets and diagnostic markers for PD. In this study, a novel lncRNA-T199678 (Lin et al., 2018) suppressed α-Syn-induced neuronal damage by targeting miR-101-3p in the α-Syn-induced SH-SY5Y cell lines (Bu et al., 2020b), thereby providing novel potential targets for PD treatment.

Taken together, this is the first report about the function of the α-Syn/lncRNA-T199678/miR-101-3p axis in PD, which provided new potential targets for the PD treatment. Although an overexpressed lncRNA-T199678 suppressed α-Syn-induced neuronal damage, intracellular oxidative stress, cell cycle dysfunction, and apoptosis only in cellular models of PD (Bu et al., 2020b), it remains unclear whether the decreased expression of lncRNA-T199678 leads to α-Syn induced dopaminergic neuron injury in animal models before clinical applications. In addition, further studies will be needed to investigate how the expression of lncRNA-T199678 is decreased in α-Syn-induced SH-SY5Y cell lines, and to clarify the downstream factors and pathways targeted by miR-101-3p.

## Author Contributions

YW and JX conceived the article. YW and XZ wrote the first draft. FC designed the figure. LC, YW, and JX reviewed and revised the manuscript. All authors contributed to the article and approved the submitted version.

## Conflict of Interest

The authors declare that the research was conducted in the absence of any commercial or financial relationships that could be construed as a potential conflict of interest.
